# *True-T* – Improving T-cell response quantification with holistic artificial intelligence based prediction in immunohistochemistry images

**DOI:** 10.1016/j.csbj.2023.11.048

**Published:** 2023-12-02

**Authors:** Yasmine Makhlouf, Vivek Kumar Singh, Stephanie Craig, Aoife McArdle, Dominique French, Maurice B. Loughrey, Nicola Oliver, Juvenal Baena Acevedo, Paul O’Reilly, Jacqueline A. James, Perry Maxwell, Manuel Salto-Tellez

**Affiliations:** a Precision Medicine Centre of Excellence, Health Sciences Building, The Patrick G Johnston, Centre for Cancer Research, Queen’s University Belfast, Belfast BT9 7AE, UK; b Sonrai Analytics, Belfast BT9 7AE, UK; c Regional Molecular Diagnostic Service, Belfast Health and Social Care Trust, Belfast BT9 7AE, UK; d Integrated Pathology Unit, Institute of Cancer Research and Royal Marsden Hospital, London SW7 3RP, UK; e Cellular Pathology, Belfast Health and Social Care Trust, Belfast City Hospital, Lisburn Road, Belfast BT9 7AB, UK

**Keywords:** Digital pathology, Computational biotechnology, Artificial intelligence, Immune, Response

## Abstract

The immune response associated with oncogenesis and potential oncological ther- apeutic interventions has dominated the field of cancer research over the last decade. T-cell lymphocytes in the tumor microenvironment are a crucial aspect of cancer’s adaptive immunity, and the quantification of T-cells in specific can- cer types has been suggested as a potential diagnostic aid. However, this is cur- rently not part of routine diagnostics. To address this challenge, we present a new method called *True-T*, which employs artificial intelligence-based techniques to quantify T-cells in colorectal cancer (CRC) using immunohistochemistry (IHC) images. *True-T* analyses the chromogenic tissue hybridization signal of three widely recognized T-cell markers (CD3, CD4, and CD8). Our method employs a pipeline consisting of three stages: T-cell segmentation, density estimation from the segmented mask, and prediction of individual five-year survival rates. In the first stage, we utilize the U-Net method, where a pre-trained ResNet-34 is em- ployed as an encoder to extract clinically relevant T-cell features. The segmenta- tion model is trained and evaluated individually, demonstrating its generalization in detecting the CD3, CD4, and CD8 biomarkers in IHC images. In the second stage, the density of T-cells is estimated using the predicted mask, which serves as a crucial indicator for patient survival statistics in the third stage. This ap- proach was developed and tested in 1041 patients from four reference diagnostic institutions, ensuring broad applicability. The clinical effectiveness of *True-T* is demonstrated in stages II-IV CRC by offering valuable prognostic information that surpasses previous quantitative gold standards, opening possibilities for po- tential clinical applications. Finally, to evaluate the robustness and broader ap- plicability of our approach without additional training, we assessed the universal accuracy of the CD3 component of the *True-T* algorithm across 13 distinct solid tumors.

## Graphical Abstract

**Figure:**
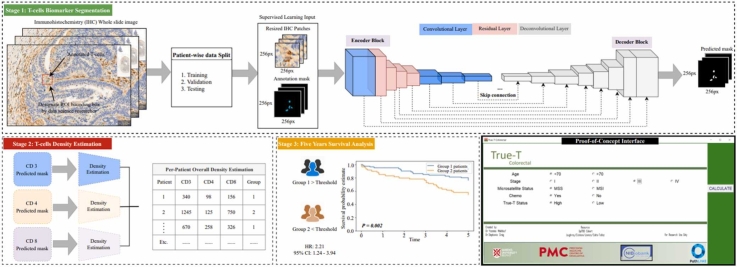

## 1 Introduction

The discovery of immune checkpoint therapy, which enhances the antitumor T-cell response to cancer Sharma and Allison [1], Sharma et al. [2], has revolutionized the field of oncology. This break-through has greatly influenced the field of cancer treatment, impacting both the present and future approaches. The integration of hybridization techniques for quantifying T-cells in cancer tissues has become widely accepted as the standard in translational and clinical research, and have been acknowledged for their substantial clinical utility in the diagnosis of colorectal cancer (CRC), both in key clinical studies Van Den Eynde et al. [3] and, more general, in international guidelines Nagtegaal et al. [4], Quezada-Mar´ ın et al. [5]. However, despit this evidence, routine utilization of T-cell quantification in everyday tissue-based diagnostics is not yet prevalent.

Histopathological slides that have been digitized and stained with immuno- histochemistry (IHC) provide a rich source of information that can be quantified and harnessed using artificial intelligence (AI), mainly via deep learning (DL) methods Singh et al. [6]; these methods have been designed to directly predict clinically significance biomarkers Srinidhi et al. [7]. Convolutional neural net- works (CNNs) with digital filters, specifically, are extensively employed to extract features from images, aiding in outcome prediction. In the realm of digital pathol- ogy, DL-based approaches are applied to various tasks, including nuclei detection, patient stratification, cell detection, and growth pattern classification using whole slide images (WSI) Aprupe et al. [8].

Abousamra et al. [9] introduced a DL framework utilizing CNN mod- els like VGG16 Simonyan and Zisserman [10], Inception-V4 Szegedy et al. [11], and ResNet-34 He et al. [12] to detect and estimate the density of tumor- infiltrating lymphocytes (TILs) in WSIs. This method effectively analyzed 23 different types of cancer, providing accurate automated TIL detection—a crucial biomarker for monitoring immune responses to diverse cancer types. Litjens et al. [13] proposed a deep CNN approach for analysis of tumour invasion front inva- sion in histopathology images; here, the CNN model captured subtle patterns of cellular invasion, providing a better understanding of cancer aggressiveness and planning treatment strategies. Matos-Cruz et al. [14], introduced a machine learning approach utilizing hematoxylin and eosin (H&E) staining to quantify the presence, abundance, and localization of tertiary lymphoid structures (TLS) as a predictive biomarker for clinical outcomes of immune-checkpoint inhibitor treatment. The authors investigated five cancer types, including bladder, breast, stomach adenocarcinoma, lung adenocarcinoma, and lung squamous cell carci- noma, using data from The Cancer Genome Atlas (TCGA). The advanced TLS model-derived features demonstrated associations with gene expression patterns and survival outcomes across various cancer types.

T-cell quantification has been suggested as part of the routine diagnostic ar-mamentarium Quezada-Mar´ ın et al. [15]. However, it is still a “prognostic” test; only when there is a clear value of this test as predictor of response (for in- stance, in Stage II & III CRC), the test will have its inherent clinical value An optimized quantification in CRC would make the test more clinically relevant, and at the same time more easily applicable Improvements in AI architectures in general, and CNN in particular, will improve the clinical applicability of T-cell analysis (for all stages), the clinical relevance (with better outcome separatetion of immune cold and immune hot groups) and a level of universal applicability of the test (across solid tumours).

In our previous research study Craig et al. [16], we provided persuasive evidence demonstrating that the quantitative analysis of chromogenic signal ex- pression of three T-cell epitopes, without using a deep learning approach, may successfully classify patients with CRC into discrete groups with notable and con- trasting clinical outcomes. The aforementioned observation demonstrated consis- tency throughout stages II-IV and is biologically associated with another essential characteristic of cancer, specifically hypoxia. Based on these findings, we hypoth- esize that incorporating a set of deep learning-based algorithms would not only enhance this clinical stratification but also yield a tool that can be more widely applicable to other tumor types. This includes the detection of biomarkers, grad- ing of malignancy, identification of invasion regions, segmentation of cell nuclei, and quantifying cell populations. The primary aim of our study is to enhance the clinical applicability of this strategy through the utilization of artificial intel- ligence (AI)-based tools, thereby advancing its alignment with real-world clinical research settings.

**Fig. 1:**
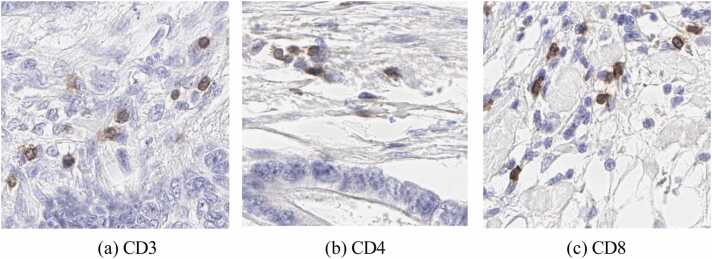
Illustration of IHC patches extracted at 40*×* magnification containing T-cell biomarkers of CD3, CD4, and CD8 in CRC. The positive cells are shown in brown cytoplasmic, and blue present the negative nuclear staining.

**Fig. 2:**
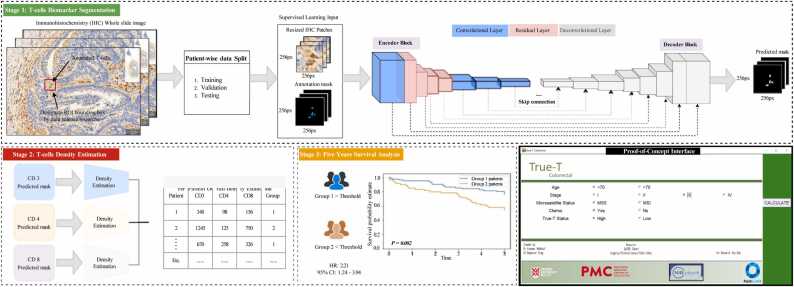
General framework of proposed *True-T*.

We have developed a novel method called *True-T*, which uses a DL-based method employing AI techniques to quantify 3 cluster differentiation antigens, representative of general (CD3), helper (CD4), and cytotoxic (CD8) T-cell func- tions in CRC using IHC images. Fig. 1 shows an example of patch images for each T-cell biomarker. The *True-T* framework has three stages: T-cells segmen- tation, density estimation, and survival rate prediction (see the general analytical framework in Fig. 2). In the first stage, we employed a standard U-Net archi- tecture with encoder and decoder layers, incorporating skip connections to refine the boundaries of T-cell segmentation. To capture spatial morphology (shape, texture, and intensity) and global feature representation for each T-cell type, we utilize a pre-trained ResNet-34 He et al. [12] model, previously trained on Im- ageNet Deng et al. [17], as a feature extractor. To achieve accurate segmen- tation, the model was trained and evaluated separately for CD3, CD4, and CD8. We measured the density of these three biomarkers in the second stage to derive a prognosis. The densities of individual T-cell biomarkers were quantified for each patient and subsequently utilized in the final stage to predict the five-year survival rate. The developed pipeline is constructed and evaluated using carefully anno- tated multi-institutional datasets from four diagnostic institutions with national accreditation, the Precision Medicine Centre Queen’s University Belfast (QUB), Oxford University Hospitals NHS Foundation Trust, Nottingham University Hos- pitals Trust, and University Hospitals Coventry & Warwickshire (UHCW). The datasets consisted of 1*,* 041 patients in total. Our gold-standard dataset was cre- ated by pathologists who provided pixel-wise accurate T-cell annotations in IHC slide images. The proposed *True-T* aims to serve as a benchmark for CRC pa- tients, and its performance was experimentally validated at each stage. Extensive ablation experiments were conducted and evaluated on an independent test set to ensure its robustness and accuracy. The study evaluated the accuracy of the CD3 biomarker across 13 different types of solid tumors, demonstrating the ro- bustness and broader applicability of the *True-T* tool without requiring additional training. Additionally, a proof-of-concept interface only for research purposes was developed to integrate the *True-T* status with other patient features such as age, microsatellite environment (MSI), and chemotherapy status. This interface provides personalized survival estimates for each patient over a five-year period, presenting the results visually through Kaplan-Meier curves and numerically as values.

## 2 Material and method

### 2.1 T-cell Biomarker dataset

**Staining and scanning:** Our models underwent training, validation, and test- ing using slides obtained from four different laboratories. All biospecimens were collected on institutional review board (IRB) approval from the respective hospi- tals or biobanks. All slides were stained in ISO 15189 (2012) quality-controlled environments using Bond Rx (QUB), Oxford, or Bond III (Nottingham University Hospitals Trust, (UHCW) platforms and scanned at 40*×* on a Leica Aperio AT2 scanner. Table 1 shows the primary antibody clones for each T-cell biomarker used by the respective laboratories. In this study, the cohort well described in Craig et al. [16] provided a larger study for independent prognostics of different T-cells including CD3/CD4 and CD8. Wagner et al. [18] and Loughrey et al. [19] leveraged the full description of metadata information.It is worth noting that, we used the same cohort as compared with Craig et al. [16].

**Table 1:** Primary antibody clones used by the respective laboratories.

T-cell Biomarker	Institution/Hospitals			
	QUB	Oxford	Nottingham	UHCW
CD3	LN10 Leica	LN10 Leica	LN10 Leica	LN10 Leica
CD4	SP35 Roche	4B12 Leica	4B12 Invitrogen	4B12 Leica
CD8	4B11 Leica	4B11 Leica	4B11 Leica	4B11 Leica

In total, we collected 1111 patients’ WSIs. Particularly, 661 cases from the Northern Ireland (NI) Biobank, as part of the Epi700 CRC cohort consisting of stage II to IV CRC patients used in numerous peer-reviewed studies to date [16], [21], [22], [23] (see ethical approval under NIB13/0069, NIB13/0087, NIB13/0088 and NIB15/0168). The other three institutions contributed 150 cases each under the general PathLAKE Consortium collaborative and ethical framework (PathLAKE 19/SC/0363). It is worth noting that appropriate consent was in place for the use of samples, images, and linked de-identified data in this research study under the ethical approvals sought from each centre, including Belfast (approval from Northern Ireland Biobank Reference NIB19–0310; NIB15–0168); in Nottingham (approval from Nottingham Health Science Biobank 15/NW/0685), in Oxford (under approval from Oxford Radcliffe Biobank 19/SC/0173) and in UCHW (un- der approval from the Arden Tissue Bank 18/SC/0180). The Northern Ireland Biobank (an HTA Licenced Research Tissue Bank with generic ethical approval.

from The Office of Research Ethics Committees Northern Ireland (ORECNIREF 21/NI/0019) to release deidentified tissues and data for research) conferred eth- ical approval for projects True-T – Improving T-Cell Response Quantification with Holistic Artificial Intelligence Based Prediction in Immunohistochemistry Images.

### 2.2 Development of robust ground-truth for T-cell biomarkers

Our automated DL-based approach aimed to design and develop an AI tool capable of scoring the density of CD3, CD4, and CD8 T-cells within regions an- notated by the pathologist on the IHC WSI. Following a model successfully used in the interrogation of immuno-oncology markers Sarker et al. [24], the ob- jective was to guarantee a robust quantification of immune CD3, CD4, and CD8 biomarkers, resulting in targeted biomarker detection.

**Fig. 3:**
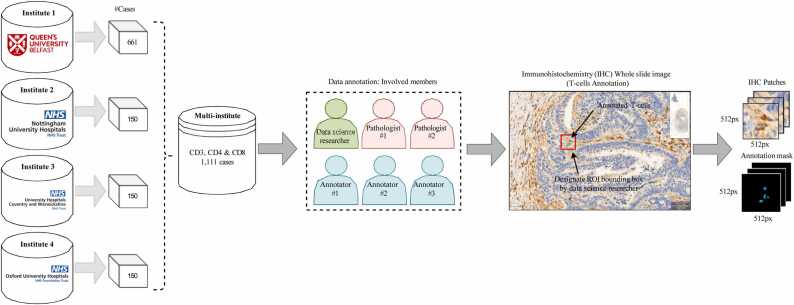
Overview of the proposed annotation data preparation pipeline. It consists of a multi- institutional dataset obtained from four different institutes in the United Kingdom. The data sci- ence researcher and pathologists’ team designed the annotation protocol followed by the three expert annotators. The corresponding patch image and annotation mask were extracted with the help of QuPath Bankhead et al. [20] software and fed to stage 1 of *True-T* pipeline.

Fig. 3 shows the general pipeline for our annotation data preparation. Our team began by creating a comprehensive reference dataset for biomarkers. We achieved this by manually annotating CD3, CD4, and CD8 positive lymphocytes with the assistance of skilled pathologists. The annotation process involved a data science researcher, three expert annotators, and two pathologists with over 15 years of experience in their field. The data science researcher initially prepared the multi-institute patient dataset into a single *.svs* project file. To help the annotators and pathologists, we designated the region of interest (ROI) bounding box of size.

**Fig. 4:**
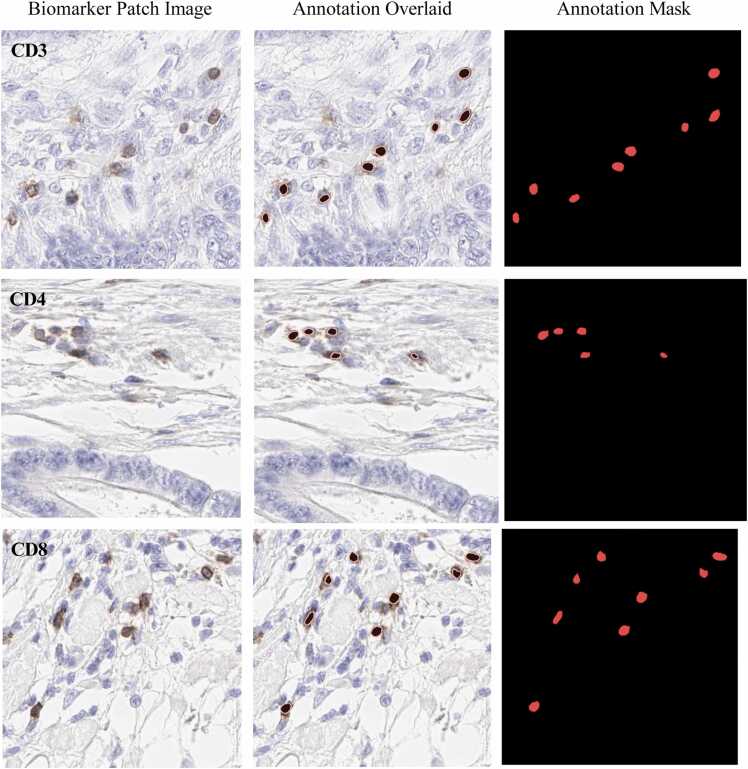
Illustration of individual T-cell biomarkers like CD3, CD4 and CD8 with their corre- sponding annotation in CRC.

512 *×* 512 pixels, allowing them to annotate the T-cells inside this region. The selection of the ROI is determined by the pathologist’s knowledge and skill and encompasses the tumour invasive margin(s) annotated on the IHC slides. Note that annotators were allowed to select any region inside the WSI. When annotators finished the annotations, an independent senior pathologist thoroughly reviewed each annotated patch to ensure quality and adjust the marking if needed. These steps were followed for each of the T-cells biomarkers. Fig. 4 shows the three T-cell biomarkers examples with their corresponding annotations in CRC.

Based on the defined criteria related to the quality of the stained and scanned slide, 1*,* 041 cases were used. Finally, the data science researcher used the open- source QuPath Bankhead et al. [20] software with version 0*.*2*.*3 to extract the patch size of 512 *×* 512 pixels with corresponding annotations and saved them into.

a *.png* image file format. The annotators used four different institutions or sites.

to annotate the 3123 ROI-defined patches containing 165860 objects (positive lymphocytes) from the 1*,* 041 patient samples. The creation of this dataset formed the foundation for training and evaluating various deep-learning architectures. We used three biomarkers of CD3, CD4, and CD8 consisting of 77555, 36969, and 51336 annotated cells, respectively. Table 2 summarizes the total number of an- notated positive lymphocytes for each T-cell biomarker.

**Table 2:** Total number of annotated positive lymphocytes for each T-cell biomarker.

T-cell Biomarker	Number of annotated cells
CD3	77555
CD4	36,969
CD8	51,336

### 2.3 True-T framework

Fig. 2 illustrates the comprehensive *True-T* framework, which comprises three key steps: T-cell biomarker segmentation from IHC, T-cell density estima- tion, and survival rate prediction.

#### 2.3.1 T-cells biomarker segmentation

For segmentation, we used the U-Net architecture, consisting of an encoder and decoder block with skip connections. The encoder block has eight layers, leveraging the ResNet-34 He et al. [12] pre-trained on ImageNet Deng et al. [17] to extract clinically relevant features like shape, texture, and intensity from patch images of T-cells. Residual blocks were employed to address the gradient vanishing problem during network training. The encoder utilized four Resnet intermediate layers, with the first layer using a 7 *×* 7 convolutional kernel to gen-erate 64 feature maps and the bottleneck layer producing 1024 feature maps with an 8 *×* 8 size.

The decoder block consisted of eight decoding layers using Transpose convo- lutions. Its main purpose was to upsample the extracted feature maps to create binary segmentation masks for each T-cell’s biomarkers. Skip connections were employed, connecting the output of each encoder layer to the input of each de- coder layer, enabling the generation of precise cell segmentation boundaries. A threshold value of 0*.*5 was used to generate the masks. Table 3 shows the best hyperparameter used to train the segmentation model. We patient-wise split our dataset into three subsets, including training, validation, and testing, with a ratio of 70%, 16%, and 14%, respectively. It is worth noting that the test set samples are kept independent and unseen throughout this process. Subsequently, we used an input size of 256 *×* 256 pixels, and in terms of patches, a total of 5536 patches for CD3, 3833 patches for CD4, and 4632 for CD8 were used. Furthermore, we normalized the data to a range of 0 *−* 1. The model was trained using the Adam optimizer with a learning rate of 0*.*0001 for 100 epochs and a mini-batch size of 16. Data augmentation techniques such as rotation up to 30 degrees and hori- zontal/vertical flipping with a probability of 0*.*5 were applied to introduce feature variability during training. To avoid the pixel imbalance, we applied the weighted cross-entropy (WCE) loss function by computing the weights of targeted T-cells and the background pixels.

**Table 3:** Summary of the best hyperparameter used to train the segmentation model.

Parameter	Value
Architecture	U-Net Ronneberger et al.[25]
Backbones	ResNet-34He et al.[12]
Batch size	16
Normalization	0 *−* 255–0 *−* 1
Optimizer	Adam
Learning rate	0*.*0001
Data augmentation	Rotation, Horizontal/vertical flipping
Epochs	100
Loss function	Weighted cross entropy

#### 2.3.2 T-cells density estimation

The segmented T-cell biomarkers CD3, CD4, and CD8 densities were calcu- lated using a connected components method. This algorithm identifies connected objects labeled as one, representing pixels belonging to each T-cell. A radius of four-pixel neighbors is considered for the connected components search. When applied to a selected ROI, cells densities are estimated for every single patch as.

follows: given that each patch of height (*h*) and width (*w*) is 512 *×* 512 *px*^2^, and based on QuPath 0.2.3 Bankhead et al. [20] each pixel area corresponds to.

0*.*25 *×* 0*.*25 µ*m*, leading to the following equation:

(1) $$Density\;per\;mm^{2}=\frac{\sum (ToC)\;\times \;10^{6}}{0.25\times 0.25\times h\times w\times no.of\;patches}$$

Where *ToC* refers to the total number of cells in the ROI of WSIs. This estimation is evaluated individually for the CD3, CD4, and CD8 slides for every patient.

#### 2.3.3 Survival rate prediction

To compute the survival analysis, we considered the outcome of each biomarker cell density. Specifically, we used receiver operating characteristic (ROC) analysis. This graphical representation helps find the optimal threshold for classifying cancer patients into two groups based on a specific measure: cell densities for each CD3, CD4, and CD8 biomarker. In this scenario, patients display distinct survival curves represented by Kaplan-Meier curves for the two groups. Separate thresholds were determined for each biomarker, enabling the division of patients into two distinct groups. A majority voting approach was employed to classify patients into two groups based on the combination of CD3, CD4, and CD8 biomarkers, referred to as *True-T* status, categorized as High or Low. More details can be found in the results section.

This framework component establishes the significance of *True-T* as a crucial indica- tor for predicting patient survival probabilities. Once this was established, we introduced a proof-of-concept interface that integrated *True-T* status with other patient features, in- cluding age, stage, microsatellite instability status, and chemotherapy treatment. The in- terface generated individual survival estimates for each patient over five years, represented graphically through Kaplan-Meier curves and as numerical values. The Cox proportional model was utilized in this process.

## 3 Results

### 3.1 Colorectal True-T: Performance

We assessed the proposed model’s efficacy by calculating its performance at two dis- tinct levels: the pixel level and the object level. At the pixel level, we determined per- formance metrics by analyzing the model’s output in comparison to ground truth anno- tations on a per-pixel basis. Five diverse metrics were employed to evaluate pixel-level performance, including accuracy, sensitivity, specificity, Dice coefficient score (Dice), in- tersection over union (IoU), and the aggregated Jaccard index (AJI). On the other hand, at the object level, we computed performance metrics by examining the correspondence be- tween ground truth annotations and the model’s output on a per-object basis, considering 4-pixel connectivity. The precision and recall scores were measured on the object level.

Table 4 demonstrates the results of T-cell biomarkers segmentation using the proposed model compared with state-of-the-art methods, such as FCN Long et al. [26], LinkNet Chaurasia and Culurciello [27], and DeepLabv3 + Chen et al. [28]. It’s important to note that we carried out distinct training and evaluation processes for the proposed and compared segmentation model for each individual biomarker, approaching each as a binary classification problem. The model achieved Dice coefficient scores of 70*.*31%, 67*.*6%, and 65*.*8% for the CD3, CD4, and CD8 biomarkers, respectively. DeepLabv3 + secured the second-highest scores for each T-cell biomarker, leveraging the extraction of multi-scale contextual information through atrous convolutions at various scales. In contrast, LinkNet recorded the lowest scores across all metrics, indicating subpar seg- mentation performance. Additionally, FCN yielded lower Dice scores than the proposed model, with margins of 3%, 7%, and 10% for CD3, CD4, and CD8, respectively. From our.

**Table 4:** Performance metrics of the proposed model comparing with state-of-the-art methods for each T-cell biomarker segmentation.

Model	Biomarker	Pixel level						Object level	
		Accuracy	Sensitivity	Specificity	Dice	IoU	AJI	Precision	Recall
FCN	CD3	95.7	75.37	97.39	67.58	58.11	54.39	71.26	66.36
	CD4	97.67	69.16	98.81	60.58	54.47	45.68	77.69	60.84
	CD8	95.77	71.86	97.80	55.20	48.38	45.06	69.50	66.21
LinkNet	CD3	95.18	68.82	98.20	65.87	54.21	53.64	68.68	62.28
	CD4	97.84	58.16	99.35	59.10	54.36	43.81	75.14	59.56
	CD8	95.86	68.72	98.19	58.03	51.02	48.29	67.47	62.79
DeepLabv3 +	CD3	95.55	75.01	97.24	68.77	51.75	53.48	73.95	68.11
	CD4	97.64	68.94	98.79	60.45	53.64	45.10	76.12	57.48
	CD8	95.87	74.22	97.97	58.54	52.29	50.65	67.86	64.62
**Proposed**	**CD3**	**98.33**	**79.27**	**98.96**	**70.31**	**60.34**	**57.57**	**78.45**	**71.21**
	**CD4**	**99.26**	**85.22**	**99.41**	**67.60**	**62.13**	**56.18**	**86.97**	**68.84**
	**CD8**	**96.94**	**80.61**	**98.99**	**65.80**	**59.66**	**57.81**	**78.35**	**75.19**

experimental analysis, we observed that the model’s performance in CD8 detection was affected by variations in staining and scanning quality from certain sources. Nonetheless, it also demonstrated substantial results as it showed a degree of generalization to other T-cell biomarkers. The model achieved notably superior results at the object level, with a precision score exceeding 78%. It effectively demonstrated a robust agreement with pathologist annotations and precisely quantified CD3, CD4, and CD8 T-cells. We also plotted the AUROC curve for CD3, CD4 and CD8 as shown in Fig. 5.

**Fig. 5:**
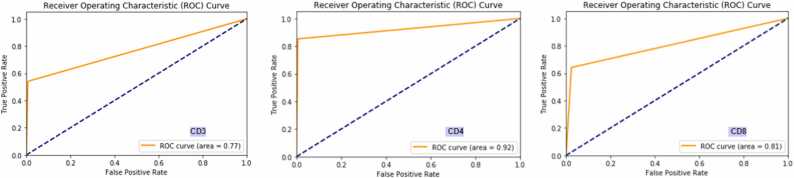
Illustration of ROC curves for CD3, CD4, and CD8.

**Fig. 6:**
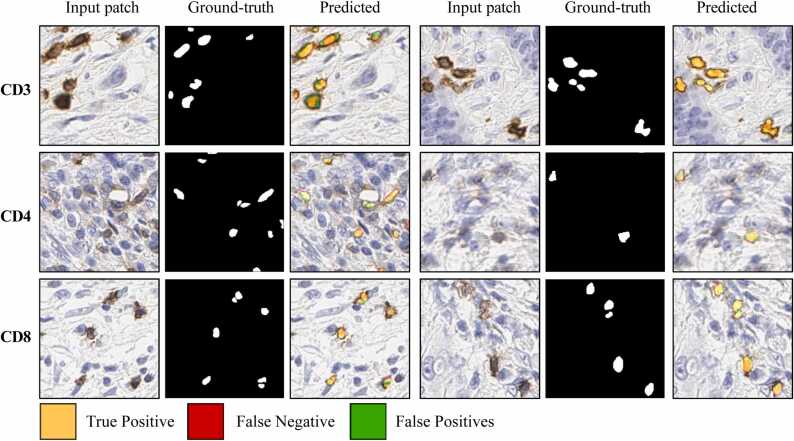
Illustration of proposed model’s segmentation results for CD3, CD4, and CD8. Exam- ples of each T-cell type were chosen, showing manual annotation and model output.

Fig. 6 shows the two examples of each biomarker type that are compared with pathologist ground-truth annotations and corresponding mask predicted by the segmenta- tion model. Notably, in the case of CD8, the boundaries of specific T-cells can be ambigu- ous and challenging to determine with precision. We provided the color maps to visualize the predicted mask against the ground truth. The colors yellow/orange, red, and green correspond to the true positives, false negatives, and false positives. Visual inspection confirmed the model’s accurate segmentation of T-cell types like CD3 and CD4, effec- tively identifying positive T-cells. The model has a high accuracy in identifying positive cells and produces minimal false positives. However, it encounters challenges in segment- ing CD8 cells due to the interconnected T-cell boundaries, leading to poor segmentation. The proposed model had difficulties in separating the connected cell boundaries. Our pri- mary objective is to calculate the density of these cells, so we are more concerned with the object-level performance rather than the pixel level. We observed that the model has achieved significantly high performance for each T-cell type.

**Fig. 7:**
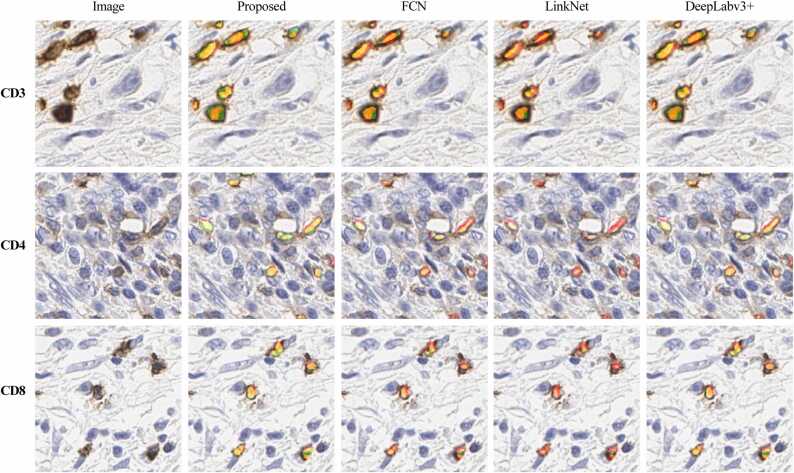
Illustration of proposed model’s segmentation results against other methods for CD3, CD4, and CD8.

On the other hand, we also provided the proposed model qualitative comparison with existing state-of-the-art segmentation methods. Fig. 7 shows the examples of predicted masks generated using the proposed model compared with other segmentation methods. From the visual inspection, we found that existing compared methods produced weak segmentation with more false positives (shown in green) that lead to overall poor per- formance. However, the proposed model delineates the cell boundaries precisely with minimal false positives.

Considering the segmentation performance of the proposed model for these three biomarkers, we established a satisfactory level of confidence to extend our analysis to an independent subset consisting of 141 patients. This subset is sourced from QUB, and it is worth noting that metadata was unavailable for the slides provided by the three other in- stitutions. In this analysis, we evaluated the CD3, CD4, and CD8 densities within specific ROIs identified by our pathologists for each patient.

**Fig. 8:**
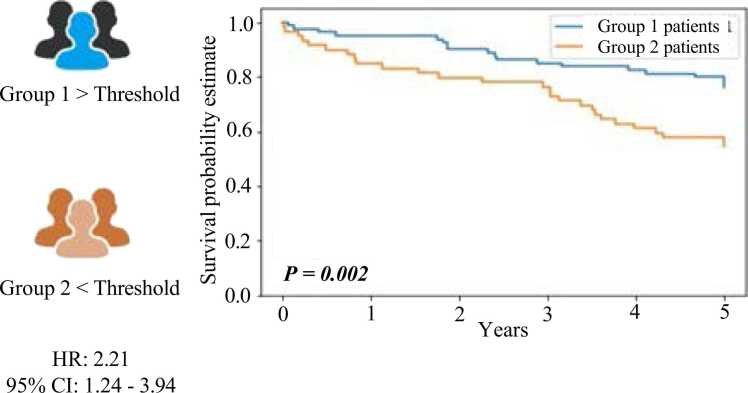
Illustration of Kaplan-Meier survival curves based on the three biomarkers with CD3- CD4-CD8 combination status.

We utilized ROC analysis to evaluate each T-cell biomarker and subsequently con- ducted a survival analysis. The goal was to determine a threshold value that could effec- tively segregate patients into two distinct groups (1 and 2) based on the available follow-up data (survival time) and the model-predicted densities. We employed a majority voting approach to assign each patient to a particular group, considering threshold values of 500 for CD3, 300 for CD4, and 700 for CD8. Patients who fell below the threshold were.

classified as having a low ”*True-T*” status (i.e., at least two biomarkers’ densities are be- low the respective thresholds), while those above the threshold were classified as having a high ”*True-T*” status (i.e., at least two biomarker densities above the respective thresh- olds). For each cancer types, the threshold values of CD3/CD4/CD8 will change due to the density scores based on each T-cell biomarker. Subsequently, we conducted uni- variate survival analysis using the Kaplan-Meier method for each group. We applied the log-rank test to evaluate the statistical significance of the survival disparities between the two groups. Fig. 8 depicts the Kaplan-Meier survival curve derived from the combined T-cell biomarker scores of CD3, CD4, and CD8. When considering combining these three biomarker scores during the majority voting step for generating the Kaplan-Meier curves, a noticeable divergence in survival outcomes became evident between patients assigned to Group 1 and Group 2. The classification into these groups was based on the opti- mal threshold value obtained by ”*True-T*.” The statistical significance of the difference in survival curves was evaluated using the log-rank test, yielding a p-value of 0.002. This p-value confirms the statistical significance and aligns with the findings presented in Craig et al. [16].

**Fig. 9:**
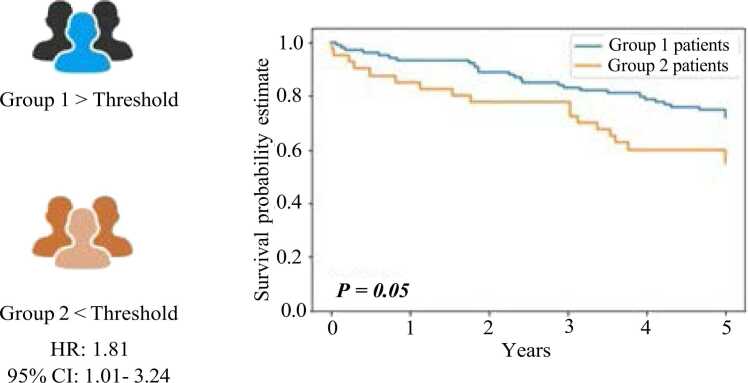
Illustration of Kaplan-Meier survival curves based on the two biomarkers with CD3- CD8 combination status.

Fig. 9 shows the survival curve considering CD3 and CD8 scores only. Given that most previous T-cell applications primarily focused on CD3 and CD8 scoring, we hy- pothesized that incorporating CD4 would offer a clinical advantage. When examining the combination of CD3 and CD8 scores alone (see Fig. 9), the log-rank test applied to the.

Kaplan-Meier curves for the two patient groups yielded a lower level of statistical signifi- cance (p = 0.05) compared to the combined inclusion of CD3, CD4, and CD8 scores. This finding aligns with the analysis presented by Craig et al. [16] and supports incorporating CD4 to provide added value.

**Fig. 10:**
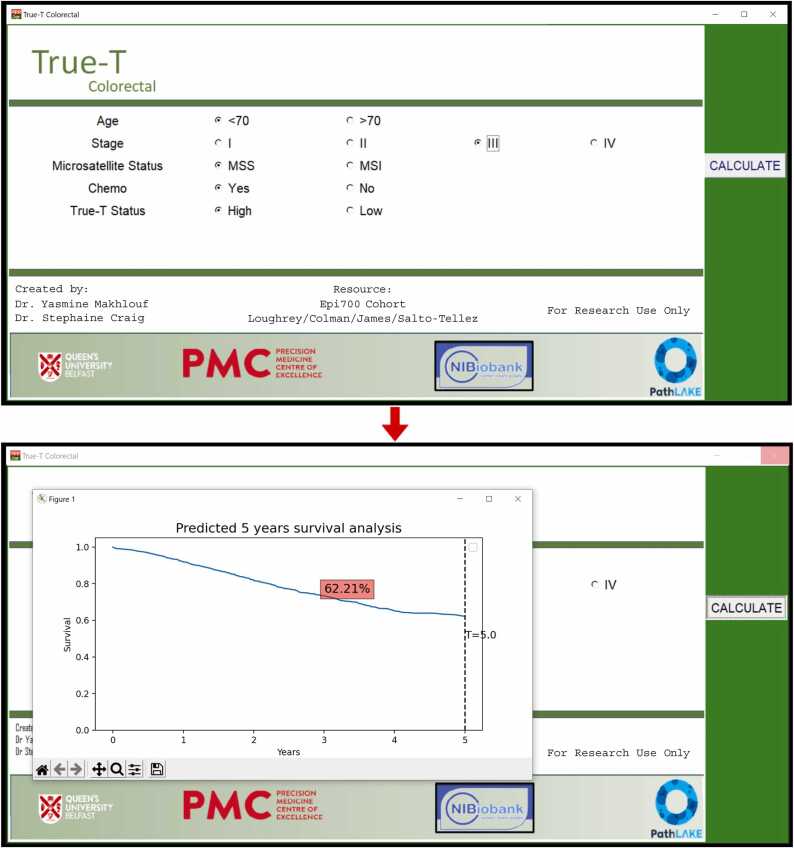
Proof of Concept interface for *True-T predictor.* This tool is specifically designed for research purposes exclusively.

We developed a simple proof of concept (PoC) interface only for research purposes viz the *True-T* predictor as shown in Fig. 10 that facilitates rapid estimation of indi- vidual patient survival over five years based on their ”*True-T*” status. The purpose of this interface is to offer a user-friendly tool for pathologists to efficiently evaluate patient prog- nosis. When a pathologist selects a patient profile, the interface presents both the survival estimate as a percentage and the corresponding Kaplan-Meier curve. The ”*True-T*” status was established based on the combined scores of CD3, CD4, and CD8, categorizing it as either low or high. Furthermore, the interface incorporated additional pertinent patient details, including age, chemotherapy status, microsatellite instability status, and staging.

### 3.2 Universal CD3 scoring

**Fig. 11:**
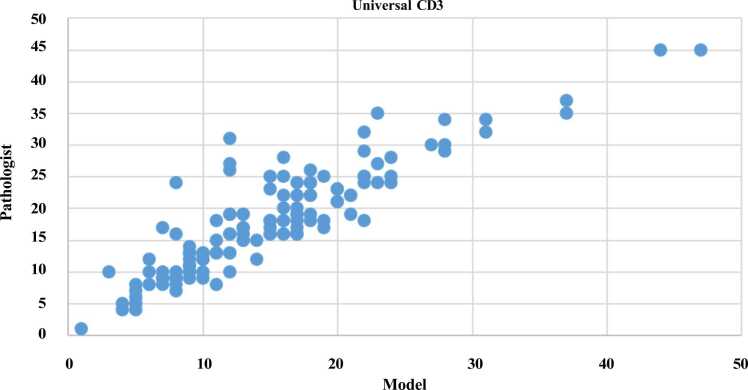
Illustration of linear correlation between the CD3 model predicted against pathologist manual scores for 13 different cancer types.

We hypothesized that a robust tool for quantifying T-cells in solid tumors might have biological relevance across a range of solid tumor types. To examine this hypothesis, we assessed the performance of our CD3 model, initially trained on colorectal cancer (CRC), on a cohort of 130 patients representing 13 distinct tumor types without any further train-

ing. For each tumor type, we selected ten patients (except for breast cancer, where we chose 20 patients), with each patient represented by a single core in a tissue microarray format. The included cancer types encompassed bladder cancer (transitional cell car- cinoma), various molecular subtypes of breast cancer, both adenocarcinomas and squa- mous cell carcinomas in lung cancer, adenocarcinomas and squamous cell carcinomas in oesophago-gastric cancer, oropharyngeal squamous cell carcinomas, ovarian serous carcinomas, pancreatic ductal adenocarcinomas, prostate adenocarcinomas, small bowel adenocarcinomas, and colorectal adenocarcinomas. Our model’s CD3 scores displayed a robust linear correlation when compared to the manual annotations provided by the pathologists. This correlation was quantified using the Pearson correlation coefficient, yielding a value of 90%. This high correlation underscores the quality and universality of our proposed CD3 model. Fig. 11 visually illustrates the linear relationship between the pathologist and model scores.

## 4 Discussion and conclusion

In the realm of potential biomarkers explored in the scientific literature, only 1% ultimately find their way into routine clinical or diagnostic use Kern [29]. This phe- nomenon can largely be attributed to the various ”reality filters” inherent in biomarker development, including the critical requirement for robust validation strategies and pre- cise quantification, as suggested by Salto-Tellez and Kennedy [30]. Leveraging AI in the analysis of protein signals in tissue hybridization tests holds the potential to introduce.

an additional level of accuracy and reproducibility. This advancement has the capacity to facilitate the inclusion of more biomarkers in the realm of clinical applicability.

The development of dependable supervised deep learning tools necessitates the estab- lishment of a robust ground truth. In our study, we developed a reference dataset for CD3, CD4, and CD8 lymphocytes in colorectal cancer cases, drawing from the expert annota- tions provided by pathologists. A team of expert annotators and two certified pathologists collaborated in creating this dataset Makhlouf et al. [31]. To maintain data quality, an independent senior pathologist conducted a thorough review of all annotations. This rigorous assessment encompassed the examination of each annotated patch employed for training and testing the AI model (as shown in Fig. 4). Our commitment to these rigorous procedures was aimed at ensuring the reliability and accuracy of the data utilized in our study.

Ensuring reproducibility is a significant challenge in the field of machine learning tools. Typically, researchers choose representative values for biomarkers and employ ad- ditional examples to construct classifiers, as documented in references such as Bankhead et al. [20]; Jhun et al. [32]. However, these conventional approaches may need to be revised to ensure robust reproducibility across diverse datasets from different institutions, primarily due to variations in section preparation and staining methods. To address this challenge, we incorporated clinical examples stained for CD3, CD4, and CD8 from four additional laboratories adhering to ISO-15189 (2012) standards. By amalgamating data from multiple sources, including different staining procedures, we aimed to account for the inherent variability in the staining process. In our study, we employed a T-cell seg-

mentation method based on the ResNet-UNet architecture to assess the performance of our proposed approach. We established a systematic process for evaluating performance metrics at the pixel, object, and case levels, resulting in high accuracy.

We assessed the effectiveness and resilience of the proposed pipeline by extending the application of the CD3 tool to different types of cancer. Our findings illustrated that well-supervised deep-learning tools can be successfully employed across a range of solid tumors. This underscores the analytical robustness and the potential for broader clini- cal applicability of our approach. Furthermore, our supervised DL development led by.

pathologists, combined with an AI approach within a quality management system, aims to enhance reproducibility and clinical utility. Our approach combines the output of multi- ple stains using DL, integrating biomarker outputs with well-established predictor factors for CRC, such as age, stage, chemotherapy status, and MSI status. This innovative ap- proach has been further supported by more recent studies Foersch et al. [33]; Chen et al. [34].

Our output matrix *True-T* identified patients’ survival in the case number available with robust prediction in CRC stages II-IV. Looking more closely at the approach taken by Foersch et al.Foersch et al. [33], there are some technical similarities but also some important differences. Firstly, our training, validation, and test cohorts consist of WSI, in contrast to the mix of Tissue Microarrays (TMAs) and WSI used in the Foersch et al. Foersch et al. [33] study. This brings *True-T* closer to the real-world clinical scenario of scoring WSI. In terms of technical approach, the Foersch et al. [33] tool, MSDLM, is more complex, utilizing the concept of attention to integrating the images of the var- ious stains (CD4, CD8, CD20, and CD68) to produce a single score of the so-called Aimmunoscore, or AIS. Our approach, while acknowledging the claims of Foersch et al. [33] that MSDLM can provide superior performance, is simpler in determining indi- vidual densities of the three markers (CD3, CD4, and CD8) prior to combining these to produce the *True-T* score.

This study also introduces a noteworthy contribution in the form of a proof-of-concept (PoC) user interface and workflow for implementing the *True-T* system. This approach aims to closely resemble the current manual scoring process, which could bring signif-icant advantages in acceptance within the pathology community. However, it is crucial to approach the utilization of these interfaces carefully. It is essential to acknowledge that these interfaces are based on cases from previous years, ensuring a sufficient clinical follow-up duration. However, this also means they might need to rely on updated ther- apeutic standards. Consequently, if constructed using local or regional data, they may not accurately reflect current national or international trends. Conversely, they may not account for regional variations if built using global data. Despite these considerations, these interfaces demonstrate the potential of a novel biomarker in a multimodal context. They offer valuable guidance for both patients and practitioners, although it is essential to remain aware of their limitations.

Over the past decade, immuno-oncology has witnessed remarkable advancements, leading to the development of a diverse array of drugs tailored to various cancer sub- types. However, a puzzling paradox exists wherein the available biomarkers associated with therapeutic response remain limited, essentially PD-L1 by IHC; MSI status by IHC, PCR, or next-generation sequencing (NGS); and tumor mutation burden by broad-based NGS analysis. In response to this challenge, we introduce a robust and straightforward method that facilitates the precise quantification of T-cells in solid tumors. This method can address the current scarcity of biomarkers and, when applied systematically to clinical trial material Salto-Tellez and Reis-Filho [35], can offer valuable insights for enhancing treatment strategies in immuno-oncology.

Traditionally, the potential clinical value of T-cells, since the early, seminal work of Galon et al. [36], is based on their quantitation. However, we also know of the different functions of similar immune cells in different solid tumours, and this may need to be taken into account in future predictive models of T-cell response.

## Authors’ contributions

Conceptualization: MST, JJ. Data Annotation: AMcA, NO, JBA, PM, MST, JJ Formal Analysis: YM. Funding Acquisition: MST, JJ Investigation: MST. Methodology Development: PM, YM, PO’R Project Administration:MST. Resources: MST, JJ, ML, SC New software development: YM Supervision: MST, PM, PO’R Visualization: YM, VS. Writing – Original Draft: MST, YM, PM, PO’R. Writing – Review and Editing: YM, MST, PM, PO’R, VS, SC,ML,AMcA,DF,JJ.

## Ethics approval

PathLAKE 19/SC/0363.

Epi700 CRC cohort: NIB19–310.

## Consent to participate

Samples and images used in this study were provided by the Northern Ireland Biobank [29] under NIB19/310. The Northern Ireland Biobank is a HTA Licenced Research Tis- sue Bank with generic ethical approval from The Office of Research Ethics Committees Northern Ireland (ORECNIREF 21/NI/0019) and can confer ethical approval for projects *True-T* – Improving T-Cell Response Quantification with Holistic Artificial Intelligence Based Prediction in Immunohistochemistry Images.

## Consent for publication

All authors have read and approved the manuscript submitted.

## Funding

This paper is supported by the PathLAKE Centre of Excellence for digital pathology and artificial intelligence, which is funded from the Data to Early Diagnosis and Precision Medicine strand of the government’s Industrial Strategy Challenge Fund, managed and delivered by Innovate UK on behalf of UK Research and Innovation. Innovation UK project reference 104689.

## Declaration of Competing Interest

Manuel Salto-Tellez is a scientific advisor to Mindpeak and Sonrai Analytics, and has received honoraria recently from BMS, MSD, Roche, Sanofi and Incyte. He has received grant support from Phillips, Roche, MSD and Akoya. None of these disclosures are related to this work..
